# 4554 Researchers’ Experiences Working With Community Advisory Boards: How Community Member and Patient Stakeholder Feedback Impacted The Research

**DOI:** 10.1017/cts.2020.280

**Published:** 2020-07-29

**Authors:** Tabetha A. Brockman, Monica L. Albertie, Noreen A. Stephenson, Sumedha G. Penheiter, Christi A. Patten

**Affiliations:** 1 Mayo Clinic

## Abstract

OBJECTIVES/GOALS: To assess researchers’ experiences working with community advisory boards (CABs) and how community member/patient stakeholder feedback impacted the research. METHODS/STUDY POPULATION: Researchers (N = 34) who presented their research to a Mayo Clinic CAB (at MN, AZ, or FL) from 2014-2017 were invited to participate in an interview in-person or by phone averaging 10-15 min. Researchers were asked “In what ways did the feedback you received from the CAB influence your research?” The validated structured 7-item interview included domains assessing potential influence that CABs had on the research: 1) Pre-research (e.g., generated ideas), 2) Infrastructure (e.g., budget preparation), 3) Research design, 4) Implementation (e.g., research recruitment), 5) Analysis, 6) Dissemination, and 7) Post-research (e.g., assist in formulating next steps). RESULTS/ANTICIPATED RESULTS: 17 interviews were completed (8 no longer at Mayo, 9 no response). Researchers presented their study to a CAB a mean of 4 years (range 3-5) before the interview. Researchers reported that the CAB had influenced their research in the following domains: 24% in pre-research, 24% infrastructure, 41% study design, 41% implementation, 6% analysis, 24% dissemination, and 18% for post-research activities. The mean total score was = 1.8 (SD = 1.7, range 0-6), of a possible range of 0-7. DISCUSSION/SIGNIFICANCE OF IMPACT: Impact of CAB feedback on the research was moderate. Ways to enhance impact could include follow-up with researchers and CAB members.

